# The first introduced malaria case reported from Sri Lanka after elimination: implications for preventing the re-introduction of malaria in recently eliminated countries

**DOI:** 10.1186/s12936-019-2843-6

**Published:** 2019-06-24

**Authors:** Vissundara M. Karunasena, Manonath Marasinghe, Carmen Koo, Saliya Amarasinghe, Arundika S. Senaratne, Rasika Hasantha, Mihirini Hewavitharana, Hapuarachchige C. Hapuarachchi, Hema D. B. Herath, Rajitha Wickremasinghe, Kamini N. Mendis, Deepika Fernando, Dewanee Ranaweera

**Affiliations:** 1 Regional Malaria Office, Moneragala, Sri Lanka; 2 Anti Malaria Campaign Headquarters, 555/5 Public Health Building, Narehenpita, Colombo 5, Sri Lanka; 3 Environmental Health Institute, National Environment Agency, Singapore, Singapore; 4 Regional Malaria Office, Badulla, Sri Lanka; 5 Regional Malaria Office, Ampara, Sri Lanka; 6 Department of Public Health, Faculty of Medicine, University of Kelaniya, Kelaniya, Sri Lanka; 7 Colombo 5, Sri Lanka; 8 Department of Parasitology, Faculty of Medicine, Colombo, Sri Lanka

**Keywords:** Introduced malaria, *Plasmodium vivax*, Prevention of re-introduction, Malaria in Sri Lanka, Reactive case detection, Entomological surveillance, Mass radical treatment, Malaria in migrant labour, Regional elimination of malaria

## Abstract

**Background:**

There has been no local transmission of malaria in Sri Lanka for 6 years following elimination of the disease in 2012. Malaria vectors are prevalent in parts of the country, and imported malaria cases continue to be reported. The country is therefore at risk of malaria being re-established. The first case of introduced vivax malaria in the country is reported here, and the surveillance and response system that contained the further spread of this infection is described.

**Methods:**

Diagnosis of malaria was based on microscopy and rapid diagnostic tests. Entomological surveillance for anophelines used standard techniques for larval and adult surveys. Genotyping of parasite isolates was done using a multi-locus direct sequencing approach, combined with cloning and restriction fragment length polymorphism analyses. Treatment of vivax malaria infections was according to the national malaria treatment guidelines.

**Results:**

An imported vivax malaria case was detected in a foreign migrant followed by a *Plasmodium vivax* infection in a Sri Lankan national who visited the residence of the former. The link between the two cases was established by tracing the occurrence of events and by demonstrating genetic identity between the parasite isolates. Effective surveillance was conducted, and a prompt response was mounted by the Anti Malaria Campaign. No further transmission occurred as a result.

**Conclusions:**

Evidence points to the case of malaria in the Sri Lankan national being an introduced malaria case transmitted locally from an infection in the foreign migrant labourer, which was the index case. Case detection, treatment and investigation, followed by prompt action prevented further transmission of these infections. Entomological surveillance and vector control at the site of transmission were critically important to prevent further transmission. The case is a reminder that the risk of re-establishment of the disease in the country is high, and that the surveillance and response system needs to be sustained in this form at least until the Southeast Asian region is free of malaria. Several countries that are on track to eliminate malaria in the coming years are in a similar situation of receptivity and vulnerability. Regional elimination of malaria must therefore be considered a priority if the gains of global malaria elimination are to be sustained.

**Electronic supplementary material:**

The online version of this article (10.1186/s12936-019-2843-6) contains supplementary material, which is available to authorized users.

## Background

Malaria was eliminated from Sri Lanka in 2012 [1] and the country received WHO certification of malaria-free status in 2016 [2]. Malaria has been moderately endemic in Sri Lanka for centuries past, and transmission was typically unstable, with seasonal-causing epidemics every 10–15 years. About two-thirds of the country, in the dry and intermediate zones (defined on the basis of rainfall) and where the principal vector of malaria, *Anopheles culicifacies*, was found are endemic for malaria [3]. The southwestern and central mountainous parts of the country have been generally free of malaria transmission.

Prior to eliminating malaria, there was a predominance of *Plasmodium vivax* infections in the country. *Plasmodium falciparum* was also present and its incidence varied over the years. From 1999 onwards, under heightened control and later elimination efforts, the malaria incidence declined steadily until October 2012 when the last case of malaria was reported. From 2008 onwards when the Anti Malaria Campaign (AMC) began classifying cases as indigenous and imported, the number of imported malaria cases increased relative to indigenous cases. Malaria in Sri Lanka on the path to elimination and post elimination is described more fully elsewhere [4, 5].

After elimination, between 2013 and 2017, 278 imported malaria cases were reported in the country [1, 6]. A high receptivity has also been recorded in some parts of the country due to the presence of malaria vectors. There have been no introduced or indigenous cases of malaria for the past 6 years post elimination until 26 December, 2018, when the first introduced case was reported, a result of local transmission. With good surveillance and a rapid response, transmission was confined to a single case of malaria. This manuscript describes the probable index case and the introduced case of malaria, which was diagnosed, and the actions taken to curtail further onward spread of the disease.

## Methods

### Study area

The country is divided into 9 provinces and 25 districts. Each province is governed by a Provincial Council. Health is a devolved subject under the jurisdiction of the Provincial Health Authority. Healthcare, both curative and preventive, is provided free of charge by central government and Provincial Councils. There is a wide distribution of healthcare facilities throughout the country with access to a health institution within 5 km.

### Malaria case and entomological surveillance

Malaria is detected by passive case detection (PCD) where diagnosis is triggered by patients seeking care for their illness from clinicians working in healthcare facilities, or active case detection (ACD). ACD is pro-active, whereby high-risk groups are screened for malaria irrespective of the presence or absence of symptoms or reactive (RCD); screening for malaria is carried out 1-km around the area of residence and amongst close contacts of an index case [7]. Malaria cases are diagnosed and managed in Sri Lanka in accordance with national guidelines [8]. Diagnosis must be based on either microscopic examination of blood smears (the mainstay of malaria diagnosis in Sri Lanka) and/or rapid diagnostic tests (RDT) before treatment. Malaria diagnostic facilities are widely available in public and private sector health institutions and facilities in the country. Polymerase chain reaction (PCR), when deemed essential for confirmation, is done at the central laboratory of the AMC of the Ministry of Health. Genotyping is requested in the event of a suspected relapse or any other relevant indication.

Since the 1930s, Sri Lanka has a strong entomological surveillance system, which has evolved based on the changing epidemiology of malaria transmission in the country [9]. Case-based entomological surveillance is initiated within 48 h of notifying a malaria case and comprehensive larval and adult surveys are carried out covering a distance of approximately 1-km radius from the location of the residence of a malaria case [7].

Methods used in confirmation of diagnosis and entomological surveillance following the detection of malaria in the individuals are described below.

### Malaria diagnosis

#### Microcopy

Thick and thin blood smears were prepared using finger-prick blood and were stained with 10% Giemsa for 10 min. Stained slides were examined using a binocular microscope under oil immersion using a magnification of 1000×. Presence of asexual blood-stage parasites was indicative of an acute infection. The parasite density was calculated from the thick smear by counting the number of parasites against 200 leukocytes (assuming 8000 leukocytes/µL), and it was expressed as parasites per µL [10].

#### Rapid diagnostic test (RDT)

Approximately 5 µL of finger-prick blood were used for RDT (CareStart TM Malaria Pf/PAN (HRP2/pLDH) Ag Combo Test). The test was interpreted according to the manufacturer’s instructions and Standard Operating Procedures prepared by AMC.

#### G6PD testing

Prior to treatment with primaquine, patients were tested for G6PD activity using the G6PD RDT (CareStart™) and the Brewers test.

### Entomological surveillance

Within 48 h of the imported malaria patient being reported, entomological surveillance was initiated by a team in and around the construction site where the index case was detected, using the following techniques: larval surveys, human landing catches (all night and partial night), indoor hand collections, pyrethrum spray sheet collections, cattle-baited trap net collections, cattle-baited hut trap collections, and window trap collections.

Two entomological teams each carried out an entomological investigation using following techniques in and around the Sri Lankan patient’s residence and the Government Hospital to which he was first admitted: larval surveys, human landing catches (partial night), pyrethrum spray sheet collections, indoor hand collections, pyrethrum spray sheet collections, cattle-baited trap net collections, and window trap collections.

### Genotyping of *Plasmodium vivax* isolates

*Plasmodium vivax* DNA was extracted from a filter-paper blood spot obtained from the imported and introduced case by using the QIAgen DNeasy Blood and Tissue kit (QIAGEN, Germany). The genetic identity of parasite strain/s was compared by using a multi-locus direct genome sequencing approach and a restriction fragment length polymorphism (RFLP) assay on *msp3α* gene as described elsewhere [11, 12]. The sequence analyses included five polymorphic loci belonging to three *P. vivax* genes, namely circumsporozoite protein (*csp*) [13], merozoite surface protein-1 (*msp1* F1, F2 and F3) [13], and merozoite surface protein 3α (*msp3α*) [11]. PCR amplification was carried out as described in the Additional file 1: Table S1. In addition, the purified PCR products of *csp* gene were cloned using the Agilent StrataClone Blunt PCR Cloning kit (Agilent Technologies, USA), according to the manufacturer’s instructions. Sixteen transfected colonies (imported; n = 12 and introduced case; n = 4) were screened by colony PCR. Sanger sequencing of amplified products (gene loci and clones) was carried out according to the BigDye Terminator Cycle Sequencing kit protocol. Raw nucleotide sequences were assembled in Lasergene version 15.0 (DNASTAR Inc., USA). Phylogenetic analyses were performed for each locus separately in MEGA7 programme [14].

## Results

### Probable origin of the introduced case

**Fig. 1:**
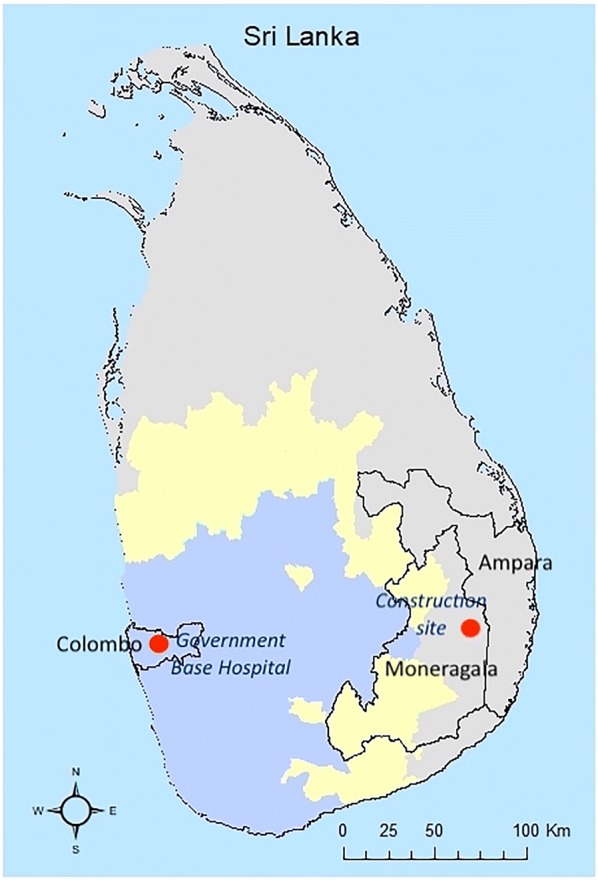
Map of Sri Lanka showing the climatic zones. Shaded in grey: dry zone where malaria was endemic; yellow: the intermediate zone where in some parts malaria was endemic and also prone to epidemics; blue: wet zone which was not endemic for malaria but very rarely epidemic prone. The district boundaries of Moneragala where the index case was resident; adjacent Ampara where some contacts were traced to; Colombo where the introduced case was resident, are demarcated. The residence of the index case and the site of transmission; the Government Base Hospital where the introduced case was first admitted to are shown by a red dot. The residence of the introduced case was near the hospital and cannot be depicted on the map at this resolution

One member of a group of 31 foreign labourers (Indian nationals), employed at a construction site in Moneragala district in the Uva Province (Fig. 1), developed clinical malaria with a *P. vivax* infection on 13 December, 2018, almost a month after arriving in Sri Lanka. The location is a rural area, which was formerly endemic for malaria. The individual, a skilled labourer, was a 24-year-old male from Uttar Pradesh, India.

Following the febrile episode, he presented to a nearby government hospital and was diagnosed as malaria on 18 December by microscopy and RDT. Ring and trophozoite stages of *P. vivax* were visible on a Giemsa-stained blood smear with a parasitaemia of 13,156 parasites/μL at the time of diagnosis (D0), and the RDT was positive for pLDH antigen. Treatment for malaria was begun immediately with chloroquine (CQ) (25 mg/kg body weight over 3 days), which resulted in a decline of the parasitaemia to 3231 parasites/μL on day 1 (D1), which further dropped to 280 parasites/μL on day 2 (D2), reaching a zero parasitaemia by the morning of the third day (D3). Following completion of the 3-day CQ therapy, G6PD testing was carried out prior to commencing treatment with primaquine (PQ) therapy (0.25 mg/kg body weight per day for 14 days) for radical cure, under supervision. This patient was classified as an imported malaria case by the Case Review Committee [15] of AMC.

**Fig. 2:**
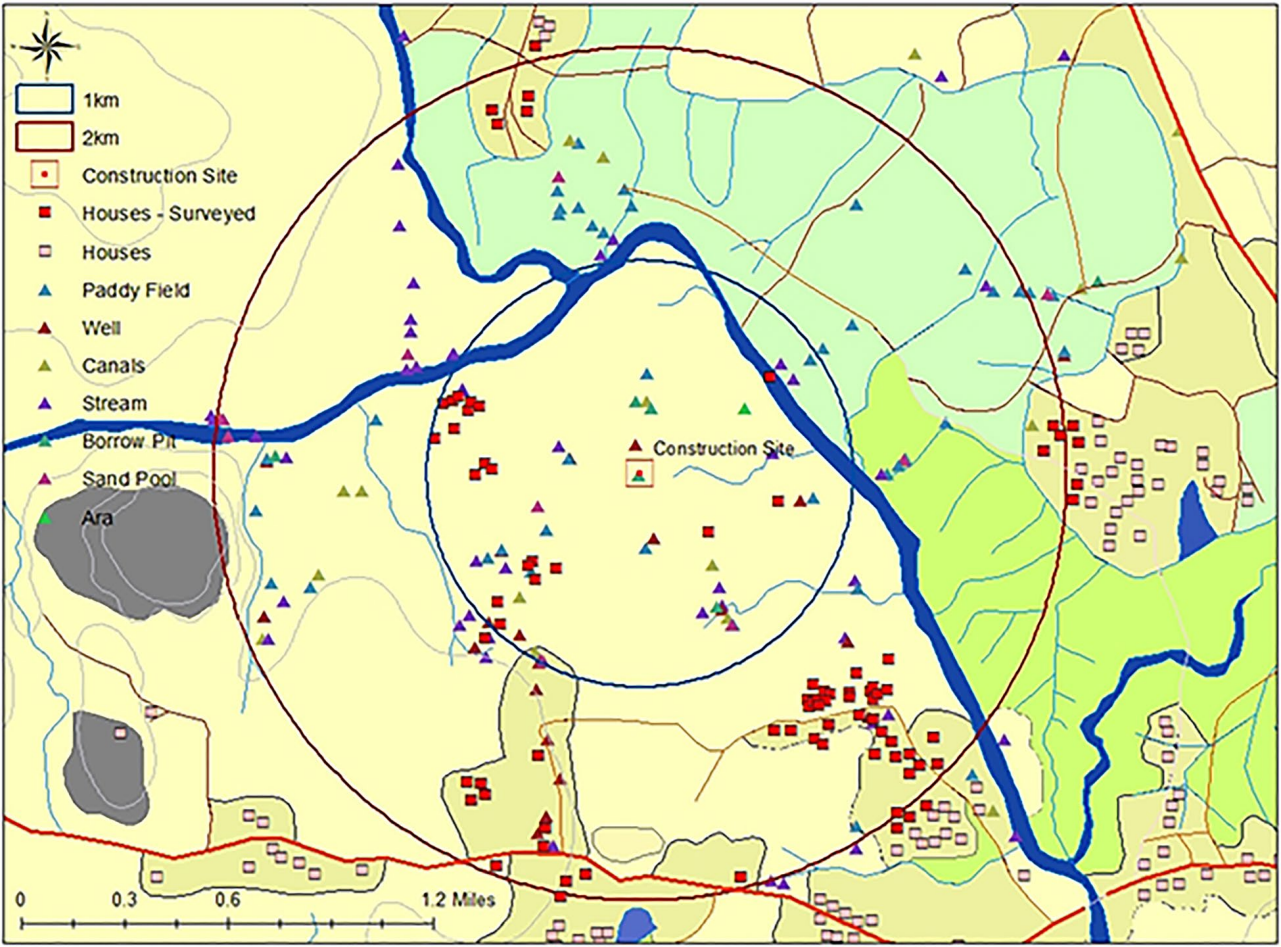
Detailed map of the area where the construction site is located (centre) where the index case was resident and where transmission took place. The zones of 1- and 2-km radius around construction site are shown in blue and red circles, respectively. All houses in the area and those which were surveyed, and the potential mosquito breeding places in the vicinity of the construction site, which were surveyed, are depicted by symbols as described in the legend. The many breeding sites that were at the construction site itself are not shown here due to the low resolution of the map

In response to this case of imported malaria, as is routine practice, AMC began case investigations within 24 h of diagnosis, followed by parasitological and entomological surveillance within 48 h at the construction site where this individual worked and resided. The workforce at the construction site was screened for malaria infection (RCD) within 48 h. Parasitological screening was also extended to residents of all houses within a radius of 1-km of the site. The houses were dispersed across two Grama Niladari (GN) divisions (the smallest administrative unit in Sri Lanka), which bordered the construction site. The site was surrounded by cane and maize plantations and there were 24 and 209 houses within a 1- and 2-km radius around the construction site, respectively (Fig. 2). Some 1190 people were screened, and none was found to be positive for malaria. Several vector-breeding places created by human activity were found at the construction site itself. High densities of *Anopheles culicifacies*, the principal vector of malaria in Sri Lanka, were found (Table 1). Vector control measures were implemented immediately at the construction site, with indoor residual spraying (IRS), larviciding, fogging and distribution of long-lasting impregnated bed nets (LLINs) to residents at the construction site. Entomological surveillance was also conducted in the area surrounding the site, and as shown in Fig. 2, several potential breeding sites were sampled in this first instance, and also later (see below).

**Table 1:** Results of the first entomological surveillance programme carried out within a 1-km radius of the construction site

Collection technique	Work output	Anopheles species (number of specimens) found
Larval survey^a^	1058 dips	*An. culicifacies* (223), *An. barbirostris* (15), *An. jamesii* (7), *An. peditaeniatus* (42), *An. vagus* (8), *An. varuna* (48)
Pyrethrum spray sheet collections	20 houses	*An. culicifacies* (3)
Indoor hand collections	5.5 man hours	*An. culicifacies* (7)
Human landing catches (indoor)	51 man hours	*An. culicifacies* (6)
Human landing catches (outdoor)	51 man hours	*An. culicifacies* (51), *An. pallidus* (1), *An. tessellatus* (1)
Cattle baited trap collections	02 traps	*An. barbirotris* (8), *An. culicifacies* (1), *An. jamesii* (2), *An. nigerrimus* (4), *An. pallidus* (3), *An. peditaeniatus* (38), An. tessellatus (5), An. vagus (14), An. varuna (61), An. aconitus (3)
Window trap techniques	06 traps	No Anophelines

^a^Potential larval breeding sites are shown in Fig. 2

### Introduced malaria case

A 45-year-old Sri Lankan national, a salesman by profession, and a resident of the district of Colombo in the Western Province (Fig. 1), which was not a malaria endemic area even before elimination, had visited the same construction site where the imported case was resident, on 30 November, 2018. He stayed there for a day and a night. On 12 December, 12 days after his return to Colombo, he developed fever followed by a productive cough for which he sought treatment, first from a private medical practitioner. Since there was no response to treatment, he presented to a Government Base Hospital in the Western Province (Fig. 1) where he was treated for a lower respiratory tract infection with amoxicillin-clavulanic acid. He discharged himself on 24 December and was admitted again to a teaching hospital nearer Colombo, where on 26 December he was diagnosed with vivax malaria. Ring, trophozoite and gametocyte stages of the parasite were recorded on a Giemsa-stained blood smear with a parasitaemia of 246 parasites/μL. He gave no history of ever travelling abroad and did not possess a valid passport, which was confirmed by the Department of Immigration and Emigration. He gave a past history of a single episode of malaria in 1996 and no history of relapses. He had had three blood transfusions in 2000. He was treated with CQ and PQ consistent with the national treatment guidelines [8], and was free of blood parasites by day 3 of treatment.

### Genotyping of *Plasmodium vivax* isolates

**Fig. 3:**
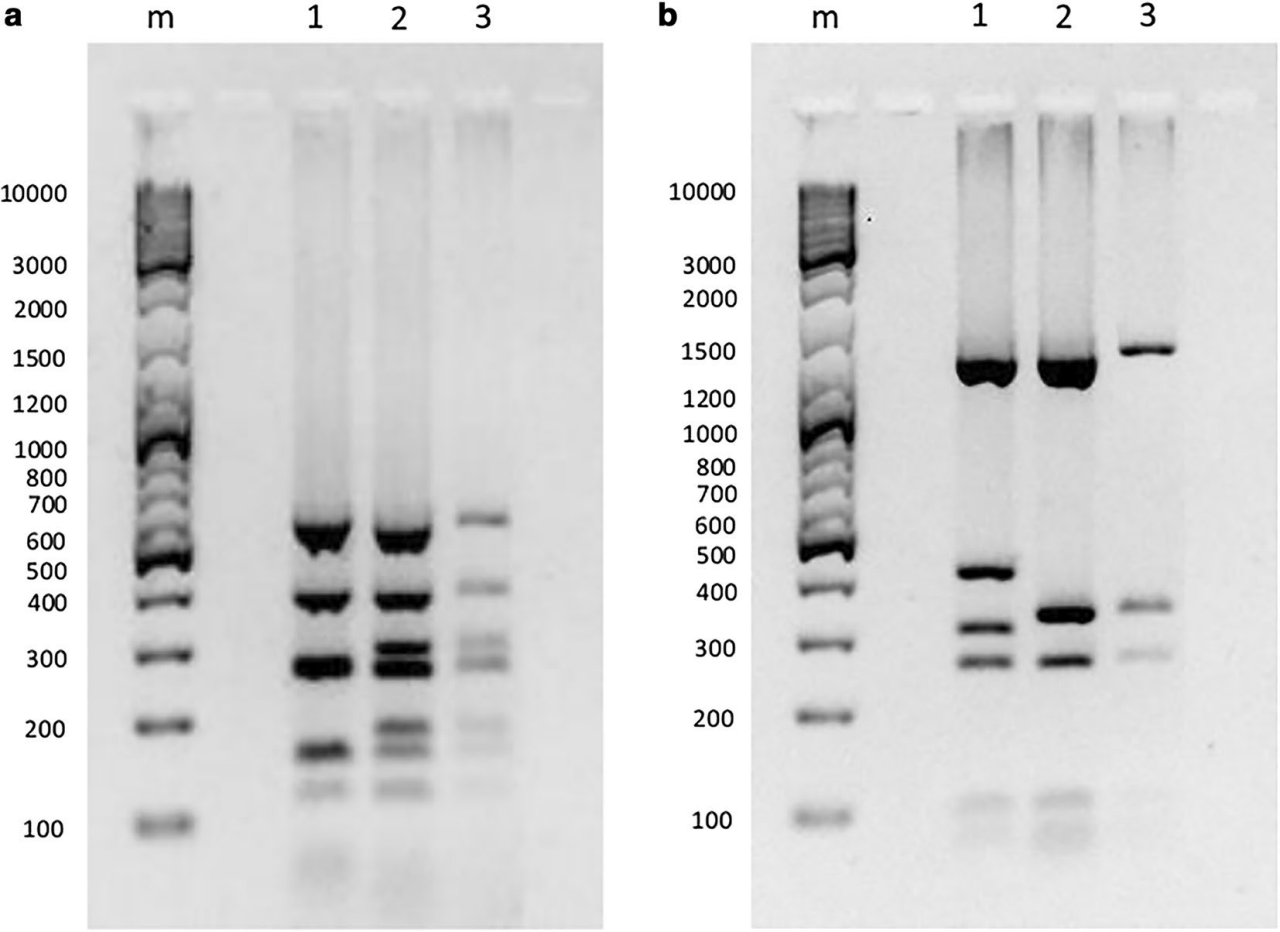
Restriction fragment length polymorphism profiles of *Plasmodium vivax msp3a* gene for imported and introduced cases. **a** Digestion with restriction enzyme *AluI.* **b** Digestion with restriction enzyme *HhaI.* DNA size marker in base pairs (bp) is indicated on the left of each panel. Faint bands obtained for the introduced case were due to low parasitaemia. 1 = Positive control; 2 = index case; 3 = introduced case; m = marker

The direct and colony PCR sequencing generated identical sequences, indicating that the parasite populations attributable to both infections were largely monoclonal. The sequencing and RFLP results (Fig. 3) showed 100% genetic identity at all loci, strongly suggesting that both patients were infected with the same *P. vivax* strain. In contrast, *P. vivax* positive control of Thai origin revealed a different RFLP pattern and sequences (Fig. 3) suggestive of close genetic relationship with Thai isolates (Additional file 2: Fig. S1; Additional file 3: Fig. S2; Additional file 4: Fig. S3; Additional file 5: Fig. S4; Additional file 6: Fig. S5), indicating the robustness of genetic comparison. The phylogenetic analyses of case sequences indicated different probable origins for each polymorphic gene, likely due to limited availability of reference sequences for each locus from different geographical regions (Additional file 2: Fig. S1; Additional file 3: Fig. S2; Additional file 4: Fig. S3; Additional file 5: Fig. S4; Additional file 6: Fig. S5). Nevertheless, *csp* and *msp1* (F1) gene sequences showed close relationship with *P. vivax* strains reported previously in India. These findings, together with other epidemiological evidence, indicated that parasites of the local (introduced) case were highly likely to be an introduction from India.

### The response to the introduced case

The response to the introduced case by AMC headquarters and the Regional Malaria Officer (RMO) was immediate and focused primarily on the site where transmission was thought to have taken place, Moneragala district, and also where the introduced case was resident in Western Province. Teams were mobilized rapidly from headquarters and the other districts to assist RMOs of the districts to where the imported case or contacts of either case were traced. The teams included medical officers, entomologists and Public Health Laboratory technicians (PHLTs) from AMC headquarters and RMOs from other districts. Vector control at the construction site that had commenced on 21 December in response to the imported case (and before the introduced case was detected) was extended and continued so as to ensure that vector densities were reduced (Fig. 2). LLINs, which were provided to all residents at the construction site earlier, were extended to residents of the surrounding villages. IRS continued at the construction site in which the imported case was detected. Vector surveillance and control continued for 8 weeks owing to the presence of a potential parasite reservoir in the group of Indian labourers and the possibility of any prevailing infected mosquitoes. Vector surveillance was also carried out in Western Province (where malaria was not endemic previously) from 27 to 31 December around the residence of, and the hospital to which the introduced case was first admitted. Although several anopheline species, some of which are considered secondary vectors of malaria in Sri Lanka, were detected in larval surveys, no *An. culicifacies* mosquitoes were detected in these surveys. Neither were adult anophelines detected by any of the techniques used.

Parasitological surveillance was strengthened and continued. All individuals who had visited the construction site were traced (contact tracing) to their current places of stay in the country and tested for malaria. RCD was carried out in each of the other districts that had been visited by the contacts. For example, three workers employed by the construction site originated from the adjacent district Ampara, which was endemic for malaria before elimination (Fig. 1). The RMO of Ampara district traced these individuals to three Medical Officer of Health (MOH) areas (sub-district health administrative areas) within this district and carried out mobile malaria clinics to screen 1584 individuals from the vicinity of the residences; none was tested positive for malaria.

The following measures were taken in the district/s where the imported and introduced cases were diagnosed and to which the contacts were traced: (a) awareness campaigns were carried out by house-to-house visits and using public address systems to alert the residents and the local population of the possibility of malaria transmission, and informing them to seek medical treatment and testing for malaria in the event of a febrile episode; (b) healthcare institutions in both public and private sectors were visited and their staff were educated on the possibility of malaria infections occurring; (c) the malaria diagnostic capacity was maximized at all health posts (both private and public sectors). Against a backdrop of a high general alert on malaria throughout the country, a circular was issued by the Director General of Health Services alerting medical staff of the case of introduced malaria, and encouraging the testing of all fever patients for malaria within the health institutions in these districts. Thereby, routine screening of all fever patients for malaria in health institutions was initiated. This was further complimented by a short message service (SMS) to alert physicians island-wide and through media coverage to the public. Guidance on curtailing the spread of malaria was provided by the Regional Directors of Health Services and Provincial Directors of the affected districts by means of internal circulars to health staff.

Four weeks after the introduced case was detected, as an additional measure, the Indian labourers employed at the construction site were treated with CQ and PQ at curative and anti-relapse doses, respectively, after obtaining written informed consent. None of them (n = 30) was positive for malaria on blood screening at baseline. All were tested for G6PD enzyme activity and radical treatment was provided as directly observed treatment only on those who tested normal for G6PD activity on both tests (n = 23) (in preparation for publication). It was ensured that medical facilities were easily accessible in the event of an acute haemolytic event [16, 17]. The subjects were closely monitored for adverse events and no serious adverse events were detected.

## Discussion

It is very likely that the Sri Lankan national acquired the *P. vivax* infection during his overnight stay at the construction site where the imported malaria patient resided, because he developed fever 12 days after his visit there. Irrespective of whether the initial fever was due to malaria, or the malaria infection developed subsequent to the respiratory tract infection at some point in time before 24 December, this would be consistent with the incubation period of the parasite. There had been no cases of introduced or indigenous malaria reported from anywhere else in the country since malaria elimination, nor had there been any other imported cases in this area; the patient had not travelled to a malaria-endemic country overseas; all this led to the conclusion that the Sri Lankan national acquired the infection at the construction site. Even though the patient gave a past history of malaria (in the absence of any supportive documentation to indicate the species), this being a relapse was highly unlikely because his anecdotal malaria event dates back two decades.

The Sri Lankan national visited the site from where it is believed he contracted the infection on 30 November 2018, which was before the imported malaria patient who was resident there reportedly developed symptoms. It is likely that the latter had a sub-clinical malaria infection, which was infectious to mosquitoes even at that time, and manifested clinically several days later. He was a resident of Uttar Pradesh, India, which is highly endemic for malaria [18]. Several such asymptomatic malaria infections have been detected among foreign workers and refugees [19] in the past few years. It has also been found that those who have been negative for malaria on screening develop symptomatic infections at various times, weeks to months after their arrival in the country on follow up, indicating that they harboured asymptomatic and/or sub-microscopic blood infections, or in the case of *P. vivax*, even dormant liver stages.

The genetic analyses of *P. vivax* strains obtained from both cases showed an identical match at five polymorphic gene loci, suggesting a strong molecular epidemiological link between the imported case and the infection in the Sri Lankan national. The *csp* and *msp1* (F1) gene sequences showed close relationship with *P. vivax* strains reported previously in India. The collective body of evidence points to the latter being an introduced case of malaria resulting from local transmission of *P. vivax*: the source of the mosquito infection, and the index case being the imported malaria patient from India who was resident at the construction site.

There was a delay of either 14 days or fewer, from the time the introduced case developed febrile symptoms to the time at which he was diagnosed as malaria, depending on whether the initial fever was the beginning of the malaria infection, or if malaria symptoms developed subsequent to a respiratory tract infection for which he was treated at the hospital. He had visited a private health care provider before presenting at the first government hospital, and had not been tested for malaria by either, but had, instead, been investigated and treated for a respiratory infection at the hospital. As reported earlier, one of the main challenges in the post-elimination phase of malaria is that, being a rare disease in Sri Lanka now, malaria tends to be overlooked by clinicians as a cause of fever in favour of other highly prevalent infections, such as those of viral and bacterial origin. However, in this case the failure to test for malaria may have been because the patient gave no history of overseas travel. The low parasitaemia of 246 parasites/μL, which he had at the time of the malaria diagnosis at the second hospital he presented to, is likely to have been due to the partial effects of medications, particularly the antibiotic that he had received during the past 14 days. The delay in diagnosis of the introduced case, though quite unacceptable, was fortunately not associated with a risk of onward transmission because the principal malaria vector is rarely found in Western Province, as was confirmed by the entomological surveillance carried out where he resided and in the vicinity of the Government Base Hospital where he was an inpatient.

The index case and the introduced case were diagnosed and treated radically. This, along with the thorough case investigation and a very rapid and thorough response (in terms of case and entomology surveillance, RCD, appropriate vector control and increasing clinician awareness), would have prevented the occurrence of any further cases, and the event was confined to a single introduced case.

Rapid response teams were mobilized from both AMC headquarters and other districts. While the parasitological and entomological activities continued in Moneragala district in relation to the index case, parasitological and entomological surveillance was conducted in Western Province, where the introduced case resided. Similar case surveillance activities were carried out promptly in all other provinces to where contacts of both cases were traced. The response to the cases by way of raising the awareness of the public and the medical professionals in the country was important because 6 years after malaria elimination clinicians are no longer familiar with the disease, and malaria is not being considered as a high probability in the differential diagnosis of febrile illnesses. Overall, the experience tested the capacity and the efficiency of the surveillance and rapid response system in Sri Lanka in the prevention of malaria re-introduction phase.

Given the heavy presence of imported labour in the country, mainly from India, and from other neighbouring malaria-endemic countries, and the high malaria receptivity in parts of Sri Lanka as documented here, the occurrence of an introduced case is not entirely unexpected. Nevertheless, this event highlights several important aspects relating to the prevention of re-establishment of malaria in the country and elsewhere.

Active case surveillance by regular screening of foreign worker groups for malaria, which is highly labour intensive, is a necessity, but may not be sufficient as a measure to reduce the risk of transmission in malaria-receptive areas. This is because many who are aparasitaemic at the time of screening by microscopy and RDT, but become clinically and parasitologically patent at various times subsequently, indicates that they harbour latent blood or liver stage infections [19]. Radical mass treatment of such groups of foreign labour from endemic countries who are likely to be infected on account of their previous exposure may need to be considered as a strategy by AMC in areas of high receptivity.

The continuing role and extent of entomological surveillance in the country after malaria elimination, has been the subject of much debate over the past few years. However, this episode of local transmission and its effective management to prevent further transmission serve to highlight the importance of entomological surveillance. It was the routine entomological surveillance carried out in response to the imported case on 21 December (which was before the introduced case was detected on 26 December) that alerted the AMC to the high transmission risk at the site and led to the commencement of vector control. This would have averted any further transmission that could have occurred during the period between the detection of the two cases and possibly prevented an outbreak, not just at the site, but also in other parts of the country because there was considerable movement of labour between the construction site and other districts. Historically, the resurgence of malaria in Sri Lanka (then Ceylon) in 1963 after near elimination, which led to endemic malaria for the next 50 years, was traced to similar events. From a focus of transmission in a gem-mining city, where receptivity had been high, people carried malaria infections to various parts of the country [20]. The current experience re-affirms the critical role of entomological surveillance for the purpose of estimating and mitigating the risk of malaria re-establishment under the continuing threat of imported malaria. It also emphasizes the need to now shift the focus of entomological surveillance from sentinel sites (which were used prior to malaria elimination) to vulnerable sites such as those areas inhabited by groups of foreign labour from endemic countries.

The prevention of malaria re-introduction programme is structured such that AMC is a central government body which provides technical guidance to Regional Malaria Offices, which are the implementing agencies in a devolved provincial authority. The Regional Malaria Offices have dedicated staff for malaria work and also undertake dengue control activities. Maintaining this structure even after elimination and malaria-free certification enabled the AMC to mount a rapid and effective response. A Technical Support Group comprising independent experts and Ministry of Health officials and chaired by the Director General of Health Services meets every 2 months and keeps the Ministry aware of malaria in terms of ensuring availability of resources in such an eventuality [15]. In addition, the AMC publishes the number of imported malaria cases reported in the country in the monthly newsletter of the Sri Lanka Medical Association, which is widely read by clinicians to keep them updated on the situation. These measures were key to the rapidity and intensity of the response mounted.

The occurrence of this case was a test for the AMC and the Provincial Health System on their surveillance and response preparedness and capacity. It also highlighted, among others, the critical need to maintain adequate emergency stocks of malaria commodities: insecticides, diagnostics and medicines, even though their expiration dates may often be reached before they can be consumed on account of the rarity of use. The introduced case was detected at the year-end holiday period when non-essential administrative operations were at ebb. And so, the effective response was mounted amidst a host of administrative and logistical challenges. The scale of operations conducted by the AMC in collaboration with the provincial health system in this instance is laudable, and will need to be maintained if Sri Lanka is to remain malaria-free. It may, however, also raise the question of cost and sustainability of such a surveillance and response system. Studies have shown that the cost of sustaining an effective surveillance and response system for malaria is far less, in economic terms alone, than that if malaria were to return [21]. When health and development costs of malaria are computed the return on an investment to prevent malaria re-introduction would be far greater.

Few countries, which have recently eliminated malaria and several that are progressing to elimination in the next few years, are in the tropical belt where receptivity remains high as in Sri Lanka [22]. This case is a reminder that the global malaria elimination drive will make little sense unless investments are made to prevent the re-establishment of the disease from countries that achieve elimination. Population movement of the kind and magnitude that impose a high risk of re-introducing malaria tend to be highest within rather than outside of regions of the world. This is due to close social, cultural and economic ties that exist among neighbouring countries. Even Sri Lanka, being an island nation, is not exempt from rampant legal and illegal migration from countries in the region, carrying with it the risk of malaria importation as shown here. Many countries in Asia (Bhutan, Nepal, Timor Leste), Middle East (Saudi Arabia, Iran) and in the Americas (e.g., Costa Rica, Suriname) [23], which are on track to achieve elimination in the next few years, share porous land borders with highly malaria-endemic neighbouring countries. The threat of re-introduction in those situations would be even greater than the one described here, and measures to mitigate that risk would be paramount. As this case serves to illustrate, it is imperative that elimination of malaria is pursued countrywide, but as a regional goal, it being the only sustainable outcome until such time that malaria eradication is achieved.

## Conclusions

Six years after malaria elimination, an imported case of vivax malaria in a foreign worker (index case) led to the first case of introduced malaria in a Sri Lankan national. An effective surveillance and response system limited transmission, and prevented further spread. Entomological surveillance data pre-empted vector control activities at the site of transmission even before the introduced case was detected. This, together with contract tracing and RCD may have averted potential outbreaks. The system of case and entomological surveillance and response needs to be sustained in this form, at least until the Southeast Asian region is free of malaria. With a high degree of movement of people within neighbouring countries in the region the risk of imported malaria remains high, carrying with it the risk of re-introducing malaria. Several countries that are on track to eliminate malaria in the coming years would be in a similar situation of receptivity and vulnerability. Regional elimination of malaria must be considered a priority goal if gains in current global malaria elimination are to be sustained.

## Additional files

**Additional file 1: Table S1.** Amplification of gene loci by the polymerase chain reaction.

**Additional file 2: Fig. S1.** Phylogenetic analysis of the *csp* gene. The maximum-likelihood tree was constructed based on the general time-reversible model with gamma distribution by using case sequences and those retrieved from GenBank (848 bp). The case sequences are highlighted in red, whereas the sequence obtained for the positive control is highlighted in blue. Figures on branches are bootstrap values. Only bootstrap values more than 70% are shown on the nodes.

**Additional file 3: Fig. S2.** Phylogenetic analysis of the *msp1* gene (F1). The maximum-likelihood tree was constructed based on the general time-reversible model with gamma distribution by using case sequences and those retrieved from GenBank (1178 bp). The case sequences are highlighted in red, whereas the sequence obtained for the positive control is highlighted in blue. Figures on branches are bootstrap values. Only bootstrap values more than 70% are shown on the nodes.

**Additional file 4: Fig. S3.** Phylogenetic analysis of the *msp1* gene (F2). The maximum-likelihood tree was constructed based on the general time-reversible model with gamma distribution by using case sequences and those retrieved from GenBank (359 bp). The case sequences are highlighted in red, whereas the sequence obtained for the positive control is highlighted in blue. Figures on branches are bootstrap values. Only bootstrap values more than 70% are shown on the nodes.

**Additional file 5: Fig. S4.** Phylogenetic analysis of the *msp1* gene (F3). The maximum-likelihood tree was constructed based on the general time-reversible model with gamma distribution by using case sequences and those retrieved from GenBank (255 bp). The case sequences are highlighted in red, whereas the sequence obtained for the positive control is highlighted in blue. Figures on branches are bootstrap values. Only bootstrap values more than 70% are shown on the nodes.

**Additional file 6: Fig. S5.** Phylogenetic analysis of the *msp3α* gene. The maximum-likelihood tree was constructed based on the general time-reversible model with gamma distribution by using case sequences and those retrieved from GenBank (1440 bp). The case sequences are highlighted in red, whereas the sequence obtained for the positive control is highlighted in blue. Figures on branches are bootstrap values. Only bootstrap values more than 70% are shown on the nodes.

## Data Availability

The data and material is available with the Director of the Anti Malaria Campaign.

## Abbreviations

ACD: active case detection
AMC: Anti Malaria Campaign
CS: circumsporozoite
CQ: chloroquine
GN: Grama Niladari Division
G6PD: glucose 6 phosphate dehydrogenase
IRS: indoor residual spraying
LLIN: long lasting insecticidal nets
MOH: Medical Officer of Health
MSP: merozoite surface protein
PCD: passive case detection
PCR: polymerase chain reaction
pLDH: Parasite Lactate Dehydrogenase
PHLT: Public Health Laboratory Technician
POR: prevention of reintroduction
PQ: primaquine
RCD: reactive case detection
RDT: rapid diagnostic tests
RMO: Regional Malaria Officer
RFLP: restriction fragment length polymorphism
SMS: short message service
WHO: World Health Organization
