# Supplementary material for: The first introduced malaria case reported from Sri Lanka after elimination: implications for preventing the re-introduction of malaria in recently eliminated countries
Source: Malar J. 2019 Jun 24;18:210. doi: 10.1186/s12936-019-2843-6 (PMC6591994; doi:10.1186/s12936-019-2843-6)
Supplement: Supplementary file 1 — Additional file 1: Table S1. Amplification of gene loci by the polymerase chain reaction. [file 12936_2019_2843_MOESM1_ESM.docx]

**Additional Table 1. Amplification of gene loci by the polymerase chain reaction.**

***Reaction components***

| Component | Volume (µl) | Final concentration |
| --- | --- | --- |
| 2X Phusion® Flash PCR Master Mix (Thermo Fisher, USA) | 10 | 1X |
| Primers (10 µM) | 1 | 0.5 µM |
| Template DNA | 2 | NA |
| H_2_O | 7 | NA |

NA=Not applicable

***Oligonucleotide sequences***

| Target | Primer name^¶^ | Sequence (5’🡪3’) |
| --- | --- | --- |
| *csp* | VCS-OF | ATGTAGATCTGTCCAAGGCCATAAA |
|  | VCS-OR | TAATTGAATAATGCTAGGACTAACAATATG |
| *msp-1 – F1* | VM1-N1F | CGATATTGGAAAATTGGAGACCTTCATCAC |
|  | VM1-O1R | CCACTCCATGAAACTGAAGTGTTTA |
| *msp-1 – F2* | VM1-O2F | GATGGAAAGCAACCGAAGAAGGGAAT |
|  | VM1-O2R | AGCTTGTACTTTCCATAGTGGTCCAG |
| *msp-1 – F3* | VM1-3F | CAAGCCTACCAAGAATTGATCCCCAA |
|  | VM1-3R | ATTACTTTGTCGTAGTCCTCGGCGTAGTCC |
| *msp3α* | P1 | CAGCAGACACCATTTAAGG |
|  | P2 | CCGTTTGTTGATTAGTTGC |

^¶^Oligonucleotide sequences have been described elsewhere [1, 2]. *csp*= circumsporozoite gene; *msp-1*= merozoite surface protein-1 gene; *msp3α*= merozoite surface protein 3α gene; F1-3=Fragments 1-3.

***Amplification protocol***

| Step | | Temperature | Duration | No. of cycles |
| --- | --- | --- | --- | --- |
| Initial denaturation | | 98 °C | 10 sec | 1 |
| Denaturation | | 98 °C | 5 sec | 35 |
| Annealing | *csp* | 61 °C | 10 sec |  |
|  | *msp-1- F1* | 64 °C |  |  |
|  | *msp-1- F2* | 67 °C |  |  |
|  | *msp-1- F3* | 68 °C |  |  |
|  | *msp3α* | 58 °C |  |  |
| Extension | | 72 °C | 45 sec | 1 |
| Final extension | | 72 °C | 1 min | 1 |
| Hold | | 20 °C | ∞ | |

The amplified products were visualized in 2% agarose gels and purified before sequencing by using the same primers used for the amplification of each locus.

**References**

1. Imwong M, Pukrittayakamee S, Gruner AC, Renia L, Letourneur F, Looareesuwan S, White NJ, Snounou G: **Practical PCR genotyping protocols for Plasmodium vivax using Pvcs and Pvmsp1.** *Malaria journal* 2005, **4:**20.

2. Bruce MC, Galinski MR, Barnwell JW, Snounou G, Day KP: **Polymorphism at the merozoite surface protein-3alpha locus of Plasmodium vivax: global and local diversity.** *The American journal of tropical medicine and hygiene* 1999, **61:**518-525.
