# Supplementary figures and images for: The first introduced malaria case reported from Sri Lanka after elimination: implications for preventing the re-introduction of malaria in recently eliminated countries
Source: Malar J. 2019 Jun 24;18:210. doi: 10.1186/s12936-019-2843-6 (PMC6591994; doi:10.1186/s12936-019-2843-6)

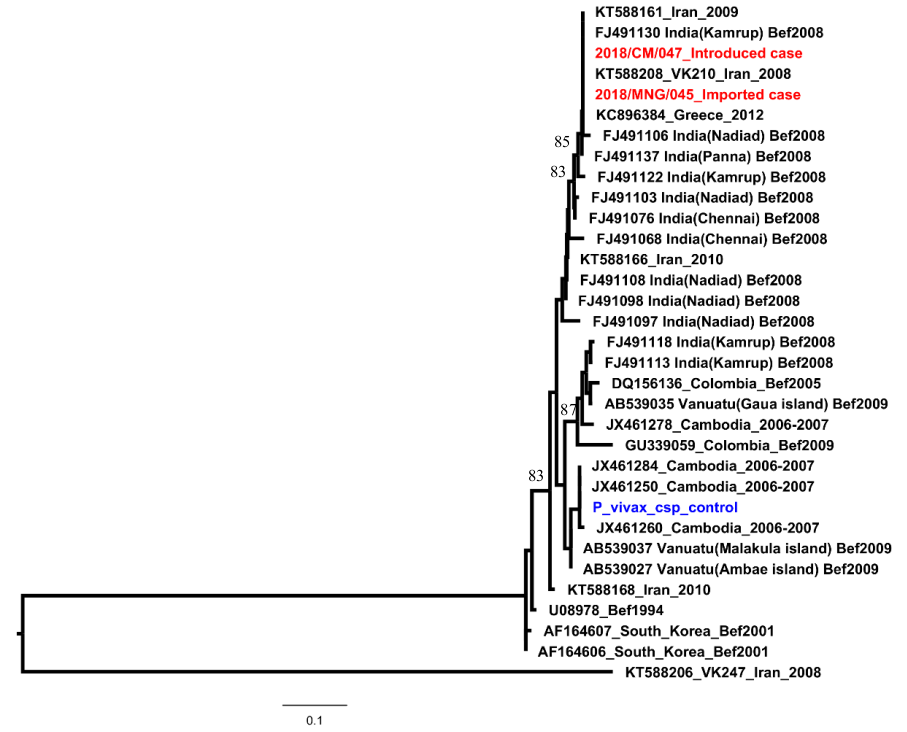

Supplement: Supplementary file 2 — Additional file 2: Fig. S1. Phylogenetic analysis of the csp gene. The maximum-likelihood tree was constructed based on the general time-reversible model with gamma distribution by using case sequences and those retrieved from GenBank (848 bp). The case sequences are highlighted in red, whereas the sequence obtained for the positive control is highlighted in blue. Figures on branches are bootstrap values. Only bootstrap values more than 70% are shown on the nodes. [file 12936_2019_2843_MOESM2_ESM.tif]

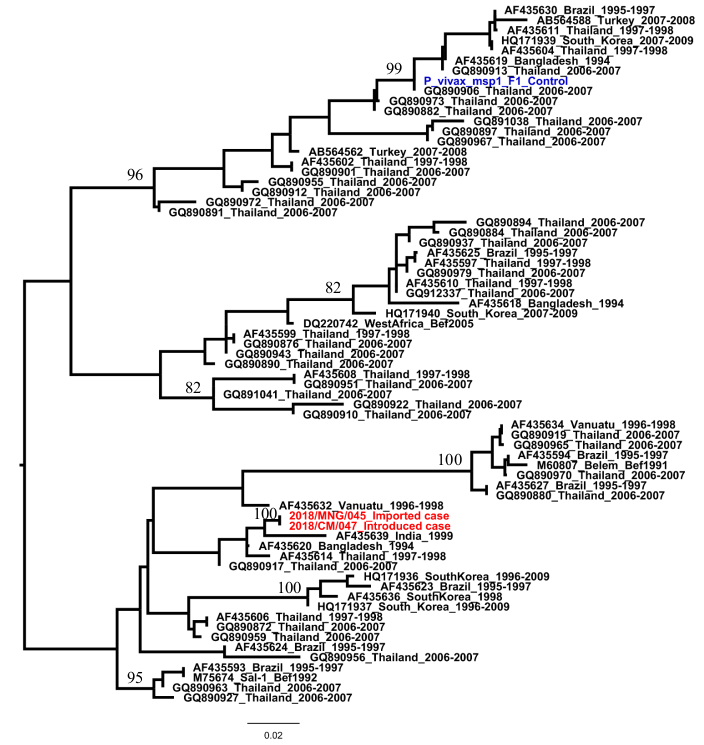

Supplement: Supplementary file 3 — Additional file 3: Fig. S2. Phylogenetic analysis of the msp1 gene (F1). The maximum-likelihood tree was constructed based on the general time-reversible model with gamma distribution by using case sequences and those retrieved from GenBank (1178 bp). The case sequences are highlighted in red, whereas the sequence obtained for the positive control is highlighted in blue. Figures on branches are bootstrap values. Only bootstrap values more than 70% are shown on the nodes. [file 12936_2019_2843_MOESM3_ESM.tif]

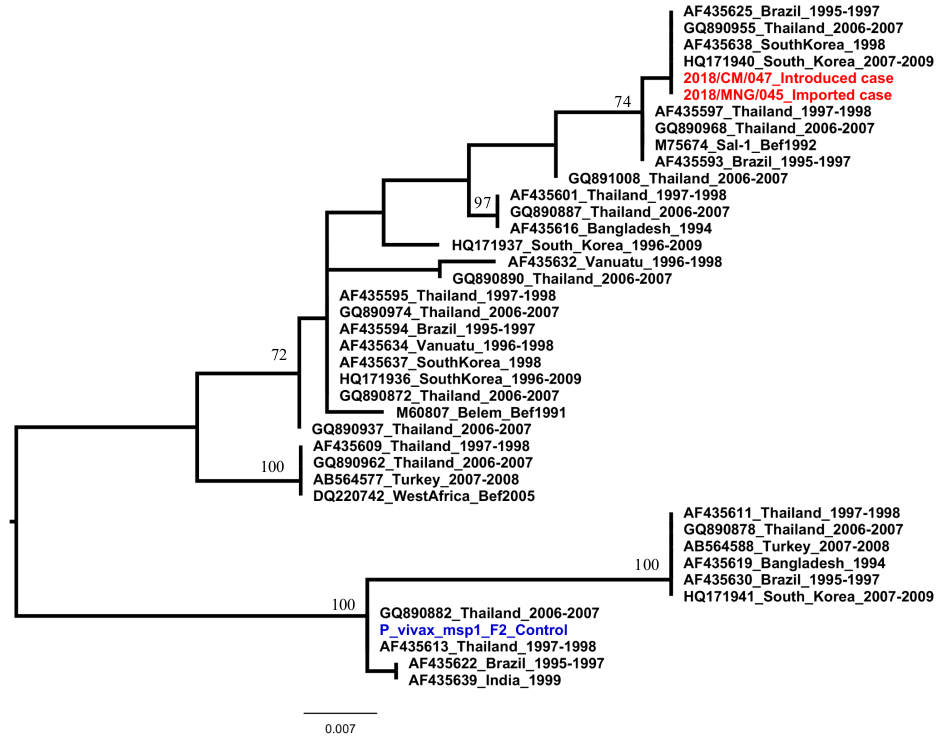

Supplement: Supplementary file 4 — Additional file 4: Fig. S3. Phylogenetic analysis of the msp1 gene (F2). The maximum-likelihood tree was constructed based on the general time-reversible model with gamma distribution by using case sequences and those retrieved from GenBank (359 bp). The case sequences are highlighted in red, whereas the sequence obtained for the positive control is highlighted in blue. Figures on branches are bootstrap values. Only bootstrap values more than 70% are shown on the nodes. [file 12936_2019_2843_MOESM4_ESM.tif]

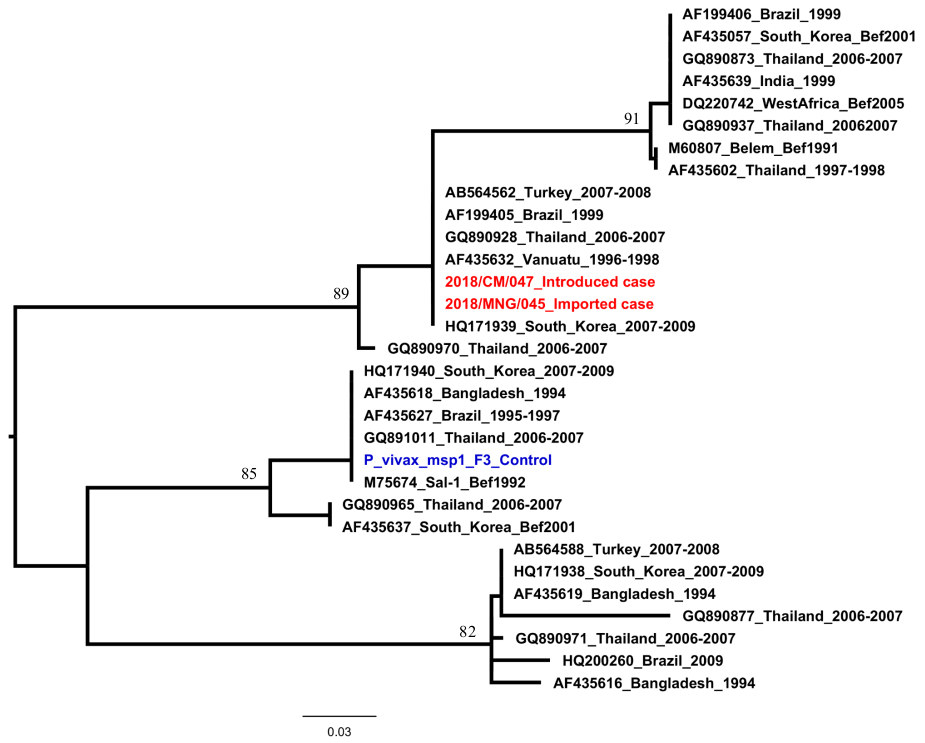

Supplement: Supplementary file 5 — Additional file 5: Fig. S4. Phylogenetic analysis of the msp1 gene (F3). The maximum-likelihood tree was constructed based on the general time-reversible model with gamma distribution by using case sequences and those retrieved from GenBank (255 bp). The case sequences are highlighted in red, whereas the sequence obtained for the positive control is highlighted in blue. Figures on branches are bootstrap values. Only bootstrap values more than 70% are shown on the nodes. [file 12936_2019_2843_MOESM5_ESM.tif]

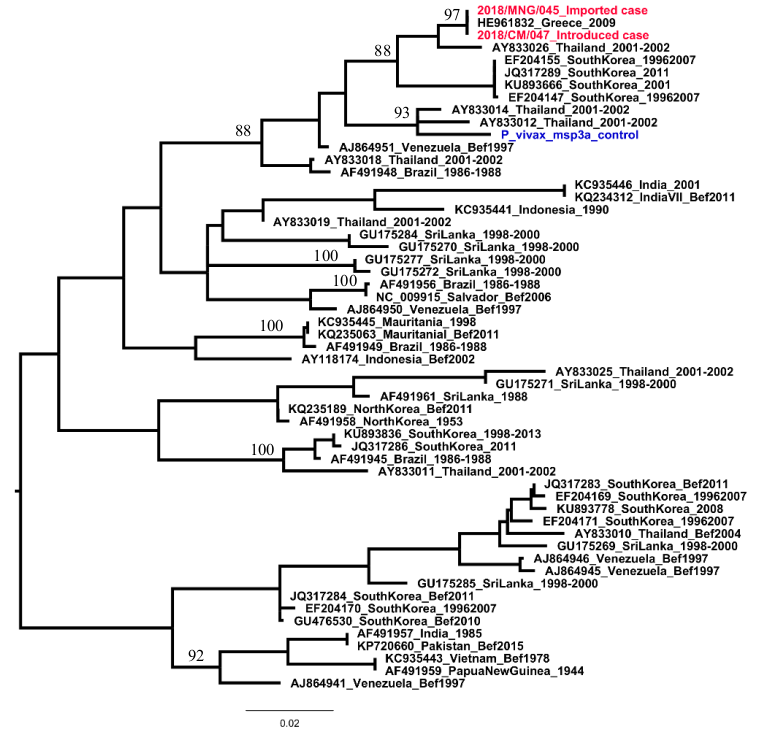

Supplement: Supplementary file 6 — Additional file 6: Fig. S5. Phylogenetic analysis of the msp3α gene. The maximum-likelihood tree was constructed based on the general time-reversible model with gamma distribution by using case sequences and those retrieved from GenBank (1440 bp). The case sequences are highlighted in red, whereas the sequence obtained for the positive control is highlighted in blue. Figures on branches are bootstrap values. Only bootstrap values more than 70% are shown on the nodes. [file 12936_2019_2843_MOESM6_ESM.tif]
